# Distinctive receptive field and physiological properties of a wide-field amacrine cell in the macaque monkey retina

**DOI:** 10.1152/jn.00484.2015

**Published:** 2015-07-01

**Authors:** Michael B. Manookin, Christian Puller, Fred Rieke, Jay Neitz, Maureen Neitz

**Affiliations:** ^1^Department of Ophthalmology, University of Washington, Seattle, Washington;; ^2^Physiology and Biophysics Department, University of Washington, Seattle, Washington; and; ^3^Howard Hughes Medical Institute, University of Washington, Seattle, Washington

**Keywords:** amacrine cell, dendritic spike, light encoding, physiology

## Abstract

At early stages of visual processing, receptive fields are typically described as subtending local regions of space and thus performing computations on a narrow spatial scale. Nevertheless, stimulation well outside of the classical receptive field can exert clear and significant effects on visual processing. Given the distances over which they occur, the retinal mechanisms responsible for these long-range effects would certainly require signal propagation via active membrane properties. Here the physiology of a wide-field amacrine cell—the wiry cell—in macaque monkey retina is explored, revealing receptive fields that represent a striking departure from the classic structure. A single wiry cell integrates signals over wide regions of retina, 5–10 times larger than the classic receptive fields of most retinal ganglion cells. Wiry cells integrate signals over space much more effectively than predicted from passive signal propagation, and spatial integration is strongly attenuated during blockade of NMDA spikes but integration is insensitive to blockade of Na_V_ channels with TTX. Thus these cells appear well suited for contributing to the long-range interactions of visual signals that characterize many aspects of visual perception.

the canonical subunit for visual processing is the center-surround receptive field in which a narrow center lies in spatial antagonism to a weaker, more diffuse surround of opposite sign. The classical center-surround computational unit extends no more than a few hundred micrometers on the retinal surface (Barlow 1953a, 1953b; Hartline 1938, 1940; Kuffler 1953). However, stimulation well outside of a cell's classical receptive field also produces clear and significant effects (Barlow et al. 1977; Ikeda and Wright 1972; Levick et al. 1965; Li et al. 1992; McIlwain 1964; Passaglia et al. 2001; Zaghloul et al. 2007), and many of these effects have been attributed to wide-field amacrine cells (Bloomfield 1996; Cook et al. 1998; Cook and McReynolds 1998; Hoggarth et al. 2015; Protti et al. 2014).

Little is known about the function of wide-field amacrine cells in any vertebrate species and particularly in primates (Baccus et al. 2008; Bloomfield and Völgyi 2007; Freed et al. 1996; Greschner et al. 2014; Ölveczky et al. 2003, 2007). The two vertebrate wide-field amacrine cells best studied physiologically are the A17 and A1, and they follow the classical pattern of collecting information over a restricted region of visual space. The A17 amacrine cell is an axonless, wide-field cell that performs important functions for visual processing under dim (scotopic) lighting conditions (Grimes et al. 2010; Hartveit 1999; Menger and Wässle 2000; Nelson and Kolb 1985). Despite its diffuse dendritic arbor, the A17 cell is involved primarily in local computations by forming reciprocal synapses, providing local inhibition back onto rod bipolar cell terminals from which the A17 receives its input. A1 amacrine cells also gather information from a restricted spatial location and then transmit that information to distant retinal targets via their extensive axonal arbors (Dacey 1989; Davenport et al. 2007; Greschner et al. 2014; Stafford and Dacey 1997).

Although the physiology is unknown, anatomical descriptions exist for many other amacrine cells in the primate retina. “Wiry” amacrine cells, studied here, have been anatomically identified in the macaque (Mariani 1990) and human (Kolb et al. 1992) retina. They lack axons and exhibit long, straight dendrites emanating ∼1 mm from the soma and stratifying narrowly within specific regions of the inner plexiform (i.e., synaptic) layer. Here we studied the biophysical and light response properties of wiry amacrine cells in primate retina with intracellular recording. The receptive fields of these wide-field cells were large, radiating structures lacking classical center-surround antagonism. The width of these receptive fields subtended >5° of visual angle. Stimulation of distal dendrites with light produced voltage responses in the soma with little degradation of signal relative to proximal stimulation. Pharmacological blockade strongly attenuated signals from distal dendrites, consistent with signal propagation via active membrane processes.

## MATERIALS AND METHODS

### 

#### Tissue preparation and electrophysiology.

All experiments were made from macaque monkey (*Macaca fascicularis*, *nemestrina*, and *mulatta* of either sex) retina in accordance with the guidelines for the care and use of animals at the University of Washington and the Washington National Primate Center. All procedures were approved by the University of Washington Institutional Animal Care and Use Committee. Retina was superfused with warmed (32–35°C) Ames medium (Sigma) at ∼6–8 ml/min. Recordings were performed from midperipheral or peripheral retina (4–8 mm, 15–30° eccentricity). All recordings were performed in whole cell current clamp with an intracellular solution containing (in mM) 125 K-aspartate, 10 KCl, 10 HEPES, 5 *N*-methylglucamine (NMG)-HEDTA, 1 MgCl_2_, 0.5 CaCl_2_, 4 Mg-ATP, and 0.5 Tris-GTP (pH ∼7.2 with NMG-OH, ∼280 mosM). Recording solution contained 0.1% Lucifer yellow for later recovery of cellular morphology (see below). Membrane potential was corrected off-line for the ∼10 mV liquid junction potential between the recording solution and the extracellular medium.

For some experiments, drugs were added to the bath solution that blocked voltage-gated Na^+^ (Na_V_) channels (TTX, 0.5 μM) and with NMDA spikes blocked [*R*-CPP 15 μM; (+)-MK-801 maleate 10 μM]. In a separate set of experiments, (+)-MK-801 maleate (1 mM) was added to the intracellular pipette solution to block NMDA receptors while the retina was bathed with regular Ames medium. This experiment was performed to avoid undesirable “circuit effects” caused by bath-applied drugs. All of these drugs were purchased from Tocris.

#### Anatomical staining and analysis.

Specimens were immersion fixed in 4% paraformaldehyde in 0.1 M phosphate buffer (PB), pH 7.4, for 30–50 min at room temperature (RT). After fixation and washing in PB, retinas were cryoprotected in a sucrose solution (30% wt/vol) and stored at −20°C until use. Polyclonal goat antibodies against choline acetyltransferase (ChAT, 1:200; Millipore AB144P; Rodieck and Marshak 1992; Yamada et al. 2003) were applied as a marker for starburst amacrine cells.

Immunocytochemical labeling was performed with the indirect fluorescence method. Retinal wholemounts were incubated freely floating for 3 days at RT or for up to 6 days at 4°C with primary antibodies in 5% normal donkey serum, 1% bovine serum albumin, and 1% Triton X-100 in PB. After washing in PB, secondary donkey antibodies conjugated to DyLight 488 (to enhance the Lucifer yellow autofluorescence) or DyLight 594 (JIR) or Alexa 633 (Invitrogen) were applied for 3 h at RT at a dilution of 1:300 (Alexa 633) or 1:500 (all remaining secondary antibodies) together with DAPI (1:2,000; Molecular Probes).

Images were taken with an Olympus FV1000 confocal microscope. Overview scans were acquired with either ×10/air or ×20/oil immersion objectives. High-resolution scanning of image stacks for *xy*-projections was performed with an Olympus UPlanSApo ×60/1.35 oil immersion objective with at least 1,024 × 1,024 pixels and a *z*-axis increment of ≤0.3 μm. For reconstructions of complete cells by “stitching,” individual image stacks from overview scans were collapsed into a single plane (*z*-projection) and manually aligned in Adobe Photoshop (Adobe Systems, San Jose, CA). The images were then loaded into ImageJ (http://imagej.nih.gov/ij/) to measure dendritic length. ImageJ was also used to perform maximum-intensity *z*- and *xy*-projections of image stacks. Brightness and contrast of the final images were adjusted with Adobe Photoshop.

#### Visual stimuli and data analysis.

Stimuli from a monochromatic oLED monitor (eMagin, Bellevue, WA) were focused on the photoreceptors through a condenser lens. The monitor illuminated a circular area ∼1.2 mm in diameter centered on the recorded cell's soma (vertical refresh, 60.2 Hz). Receptive field maps are shown with distal regions grayed out, indicating the upper limit of our projection system. Moreover, toward the outer edges blurring was apparent in the receptive fields of wiry cells, and we assume that the optical path of the projection system caused this blurring. All recordings were performed at a background in the photopic regime (quantal catch in R* cone^−1^s^−1^: L/M cone, ∼1.3 × 10^4^; S cone, ∼2 × 10^3^). Membrane voltage was sampled at 10 kHz with an ITC-18 analog-digital board (HEKA Instruments, Bellmore, NY), amplified with a Multiclamp 700B amplifier (Molecular Devices, Sunnyvale, CA), and Bessel filtered at 3 kHz. All analyses were performed in MATLAB (version 8; MathWorks, Natick, MA).

Light and dark spots, annuli, and squares were ±100% Weber contrast [(*I*_stimulus_ − *I*_background_)/*I*_background_]. To characterize the temporal kinetics of a cell, we presented a full-field spatially uniform stimulus. Stimulus contrast was drawn randomly from a Gaussian distribution on each frame (mean 0, standard deviation 0.3, range −1.0 to +1.0) and presented for 1.5–2.5 min (5,400–9,000 separate frame presentations). Resting membrane potential was subtracted from recordings, and a linear filter (*F*) was calculated by cross-correlating the stimulus (*S*) and the response (*R*).

$$\text{F}\left(\text{t}\right)=\int S^{*}\left(\text{t}-\tau \right)\text{R}\left(\tau \right)\text{d}\tau$$

where S* denotes the complex conjugate of *S* and τ is the temporal lag. After the linear filter was calculated, an input-output nonlinearity was calculated by convolving the linear filter with the frame sequence to produce a linear prediction (i.e., generator signal, *P*; *x*-axis):

P(t)=∫F(τ)S(t−τ)dτ

The prediction was plotted versus the actual response of the cell (*y*-axis) (Brenner et al. 2000; Chichilnisky 2001; Rieke et al. 1997).

The spatio-temporal noise white stimulus was presented as a grid of squares (edge length 18–45 μm, update rate 60.2 Hz, duration ∼10^5^ frame presentations). On every frame, each square was randomly black or white and uncorrelated in either space or time with other squares. We calculated a cell's spatio-temporal receptive field by cross-correlating the stimulus sequence for each square with the cell's voltage responses after subtracting the resting membrane potential. This simple calculation produced a description of how the cell filtered visual input in space and time—the spatio-temporal receptive field, which can be thought of as the optimal stimulus for driving responses in the cell. The raw spatio-temporal filters were then corrected for the vertical refresh of the monitor (i.e., the bottom of the monitor was drawn ∼17 ms before the top).

For moving stimuli, we provided values in both micrometers per second and degrees per second, using a conversion of 200 μm/° for the macaque retina at the eccentricities (4–8 mm) in which we performed recordings (Perry and Cowey 1985).

#### Computational model of wiry cells.

Wiry amacrine cell dendrites were modeled with the NEURON modeling environment (version 7.3; Hines and Carnevale 1997, 2001). The model was motivated by our anatomical data and comprised a soma (diameter 10 μm) and a single dendrite (length 1,000 μm, diameter 0.35 μm or 65 μm). Standard membrane properties were included for the soma and dendrite. Membrane capacitance was set to 1 μF/cm^2^ (Gentet et al. 2000). Dendritic values for intracellular resistivity (*R*_i_) fall in the range of 70–220 Ω·cm (Spruston et al. 2008), with lower values producing less signal attenuation. Thus we used a value of 70 Ω·cm in order to maximize the passive conductances within the dendrite.

## RESULTS

### 

#### Identification of wide-field amacrine cells.

**Fig. 1.**
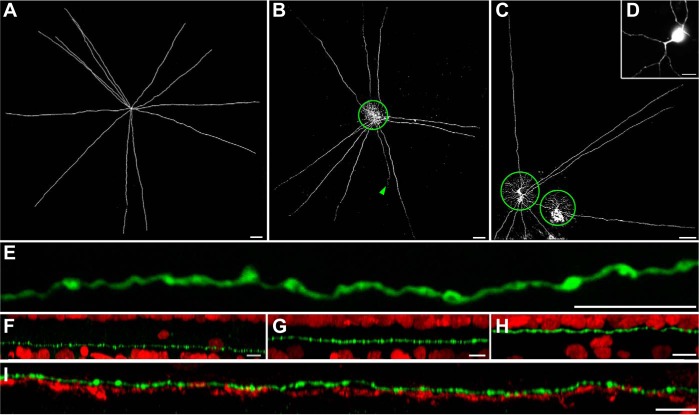
Morphological properties of wiry amacrine cells. *A–C*: *z*-projections of confocal image stacks of 2 ON (*A* and *B*)- and 1 OFF (*C*)-type wiry amacrine cell in whole-mounted retinas with overlapping parasol cells (green circles in *B* and *C*). Arrowhead in *B* points to the axon of the parasol cell. *D*: branching pattern of primary dendrites. *E*: *z*-projection of confocal image stack of a single dendrite (green) from an OFF wiry amacrine cell. The dendrite was very thin (diameter ∼0.35 μm) and exhibited putative synaptic varicosities (diameter ∼0.6–1.0 μm) along its length. *F–I*: *xy*-projections of single dendrites (green) from whole-mounted retinas, double-labeled with DAPI (red) to identify inner and outer borders of the inner plexiform layer (IPL). Wiry cells stratified at 3 distinct levels within the IPL. *I*: *xy*-projection of a single dendrite (green) from a center-stratifying ON wiry cell in whole-mounted retina, double-labeled with ChAT (red). Dendrite is located in close proximity above ON starburst amacrine cells. Scale bars: 100 μm (*A–C*), 10 μm (*D–I*).

We recorded from displaced, wide-field amacrine cells in an in vitro whole-mount preparation of the macaque monkey retina. The somata were located in the ganglion cell layer and were specifically targeted on the basis of their circular shape and small size (diameter 10.5 ± 2.6 μm, mean ± SD; *n* = 6). Dye injection into the cells revealed a sparse dendritic tree, consisting of 7–12 straight, fairly smooth dendrites (Fig. 1, *A–C*) that originated from one or two primary dendrites (Fig. 1*D*). The dendritic span from soma to tip reached an average length of 1,030 ± 197 μm (*n* = 8 cells), creating very large dendritic fields ∼2 mm (∼10° visual angle) in diameter. For reference, overlapping parasol ganglion cells had dendritic field diameters of 197 ± 20 μm (*n* = 5 cells). The amacrine cell dendrites exhibited putative synaptic varicosities along their full length but lacked spines and axonal processes (Fig. 1*E*).

The cells' dendrites stratified narrowly at one of three levels within the inner plexiform layer (Fig. 1, *F–I*). The innermost type stratified in sublamina (S)5 (*n* = 7 cells). Dendrites of the outermost type stratified in S1 (*n* = 8 cells). To further specify the dendritic stratification of the central cell, we counterstained injected amacrine cells with antibodies against ChAT, labeling starburst amacrine cells (Rodieck and Marshak 1992). The dendrites of the central-stratifying cell were found in S3/4, distal to the inner (ON) ChAT band (Fig. 1, *G* and *I*; *n* = 17 cells).

Previous morphological studies in macaque and human retinas have identified wide-field amacrine cells, termed wiry cells, with morphology and stratification patterns similar to the cells that we have recorded (Kolb et al. 1992; Mariani 1990). Thus we refer to the cells studied here as wiry amacrine cells.

#### Wiry cells comprise ON and OFF subtypes.

With the exception of the A1 amacrine cell, the physiological properties of primate wide-field amacrine cells remain unclear (Davenport et al. 2007; Greschner et al. 2014; Stafford and Dacey 1997). Here we characterized basic features of the wiry cell light responses to provide a foundation on which we can begin to understand the role these cells play in vision. The voltage responses of wiry cells to visual stimuli were recorded with a K^+^-based pipette solution in current-clamp configuration.

**Fig. 2.**
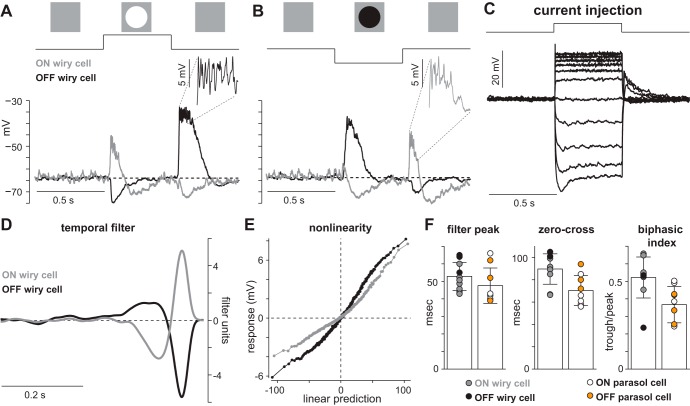
Basic light responses of wiry amacrine cells. *A*: Voltage responses of an ON-type and an OFF-type wiry amacrine cell to a 0.5-s, +100% contrast spot centered on the soma. *Inset*, 60-ms region of peak OFF cell response exhibiting high-frequency spikelets riding on top of a sustained depolarization. *B*: responses of cells in *A* to a −100% contrast spot. *Inset*, 60-ms region of peak ON cell response exhibiting spikelets. *C*: voltage response in an ON-type wiry cell to a 0.5-s family of 11 injected currents (50-pA increments from −200 pA to +300 pA). *D*: linear filters of ON and OFF wiry cells calculated with full-field Gaussian noise. *E*: nonlinear input-output functions for cells in *D*. *F*: kinetic properties derived from temporal filters of 7 ON wiry cells, 4 OFF wiry cells, 6 ON parasol cells, and 3 OFF parasol cells. Error bars denote mean and SD. *Left*: time to filter peak for the temporal filters in milliseconds. A smaller value indicates more brisk kinetics. *Center*: time to zero crossing of the temporal filter in milliseconds. A smaller value indicates more transient kinetics. *Right*: biphasic index of temporal filters. This value is the trough divided by the peak of the temporal filter.

Responses to light or dark spots centered on the soma at a photopic background (diameter 360 μm, contrast ±100%) revealed two fundamental physiological classes of wiry cells—an OFF type that depolarized to the offset of a light spot or the onset of a dark spot and an ON type that depolarized to the onset of a light spot or the offset of a dark spot (Fig. 2, *A* and *B*). The OFF functional type corresponded to the wiry cell stratifying in S1 (Fig. 1), and the ON type comprised both the S3/4 and S5 stratifying morphological types. None of the anatomical or functional properties investigated in the course of our study besides the distinct stratification levels indicated any differences between the two ON wiry types. Nonetheless, these types are likely involved in different cellular circuitries by virtue of their distinct stratification.

#### Wiry cells exhibit fast, transient kinetics.

The fundamental physiological division of wiry cells into ON and OFF types having been established, the kinetic properties of these cells were investigated by recording responses to full-field, spatially uniform Gaussian flicker (SD 0.3). A temporal filter for each cell was calculated by cross-correlating the cell's voltage responses with the stimulus sequence (Brenner et al. 2000; Chichilnisky 2001; Meister et al. 1994; Rieke et al. 1997). Wiry cells showed strongly biphasic filters with fast, transient kinetics (Fig. 2*D*) that were similar to those of parasol (magnocellular projecting) ganglion cells (Fig. 2*F*). The biphasic nature of the temporal filters is indicative of band-pass temporal frequency tuning.

#### Receptive fields of wiry cells.

The mode in which wiry cells integrate inputs across their dendrites should be reflected in the cells' receptive fields. Local dendritic integration, as is the case for A17 amacrine cells, predicts a receptive field much narrower than the dendritic field because distal inputs do not efficiently propagate to the soma (Grimes et al. 2010; Hartveit 1999; Menger and Wässle 2000; Nelson and Kolb 1985). More extensive integration, likely requiring active dendritic processes, could create receptive fields similar in extent to the dendritic fields. These predictions were tested with receptive field mapping. Spatio-temporal noise has been used extensively to measure the receptive field properties of retinal neurons (Chichilnisky 2001; Devries and Baylor 1997; Meister et al. 1994, 1995), including wide-field amacrine cells (Greschner et al. 2014; Ölveczky et al. 2003). Thus voltage responses of wiry cells to a spatio-temporal white noise stimulus were recorded. The stimulus was a circular grid of squares with a radius of ∼0.6 mm. The spatial receptive field and temporal filter (impulse response) were computed by cross-correlating the stimulus and response (Brenner et al. 2000; Chichilnisky 2001; Meister et al. 1994; Rieke et al. 1997; see materials and methods). The spatio-temporal receptive field accounted for 78 ± 5% of wiry cell response variance (*n* = 9 cells; mean ± SD).

**Fig. 3.**
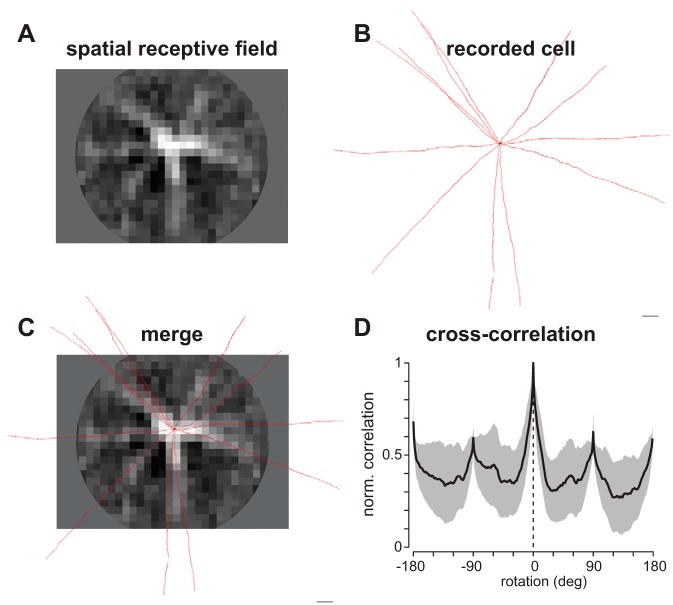
Wiry cells integrate over an extensive area of space. *A*: spatial receptive field of a wiry amacrine cell determined with spatio-temporal white noise. *B*: maximum-intensity projection of confocal images from cell in *A* injected with Lucifer yellow. *C*: merge of spatial receptive field in *A* and dendritic morphology in *B*. *D*: normalized cross-correlation of spatial receptive field and dendritic morphology as a function of rotation angle for 5 wiry cells. Plot shows means ± SE.

An example reverse-correlation map for an ON wiry amacrine cell is shown in Fig. 3*A*. Unlike the classical difference-of-Gaussians center-surround organization present in the receptive fields of many neurons, it exhibited striking long and straight excitatory regions (light colors) extending in several directions from the soma, flanked by elongated, dark surround regions. Distal areas of the receptive field are grayed out, as this represents the limit of our experimental projection system (Fig. 3*A*). Lucifer yellow (0.1%) in the recording solution allowed recovery of the cells' morphology after fixation. For example, the morphology of the cell in Fig. 3*B* revealed 12 straight dendrites radiating from the soma. To determine how the spatial receptive field related to the dendritic morphology, we manually aligned the receptive field and dendrites (Fig. 3*C*). In many cases, such as in Fig. 3*C*, the dendrites appeared to be visible in the receptive field; weaker areas of correlation may be due to artifacts from tissue fixation and mounting. However, despite all of the procedural manipulation of the tissue, the correspondence between the receptive field and morphology was striking. To assess the appropriateness of our manual alignment, we calculated the cross-correlation between the receptive field and dendrites while rotating the images relative to each other. Across cells, the cross-correlation peaked at the point of manual alignment (Fig. 3*D*, dashed line).

The receptive fields measured for wiry cells were larger in diameter than any previously described in the primate retina, eclipsing neighboring parasol ganglion cells by a factor of 10. Wiry cells transmitted signals from a distance of at least 0.6 mm to the soma (Fig. 3*A*). This value is likely an underestimate, as a radius of 0.6 mm reflects the approximate upper limit of our experimental projection system (see materials and methods).

#### Somatic recordings did not exhibit large-amplitude spikes.

Given the expected decay from passive conductances, the most parsimonious explanation for the very wide receptive fields observed is that electrogenic (action) potentials or other active membrane processes are present in wiry cell dendrites. However, typical (>25 mV) spikes were not observed to light stimulation that produced large membrane depolarizations (Fig. 2, *A* and *B*). Current injections at the soma yielded the same result. Despite membrane depolarizations in excess of 20 mV, large spikes were not detected (Fig. 2*C*). The lack of large-amplitude spikes in wiry cells was not attributable to our pipettes or intracellular solution, as sodium spikes were clearly visible from ganglion cells and A1 amacrine cells recorded with identical pipettes and intracellular solution (see Fig. 5*B*). Thus somatic recordings did not produce direct evidence for large-amplitude spikes in wiry cells.

Despite the absence of large spikes, strong stimulation of wiry cells elicited high-frequency spikelets riding on top of a sustained depolarizing envelope (see Fig. 2, *A* and *B*, *inset*). These spikelets were similar in pattern and magnitude to previously confirmed dendritic spikes in the retina (Boos et al. 1993; Cembrowski et al. 2012; Oesch et al. 2005; Werblin 1977) and other brain areas; this pattern is characteristic of dendritic NMDA spikes (Häusser et al. 2000; Major et al. 2013; Schachter et al. 2010; Smith et al. 2013). These observations support the presence of dendritic spikes in wiry cells, but the dendrites were so thin that it was not possible to test for action potentials along them directly by patch-clamp recording. However, the hypothesis that potentials are propagated these great distances as the result of dendritic spikes makes a number of predictions that can be tested by examining the temporal and spatial properties of the receptive field in detail.

#### Signal propagation along wiry cell dendrites as characterized by their spatio-temporal receptive fields.

**Fig. 4.**
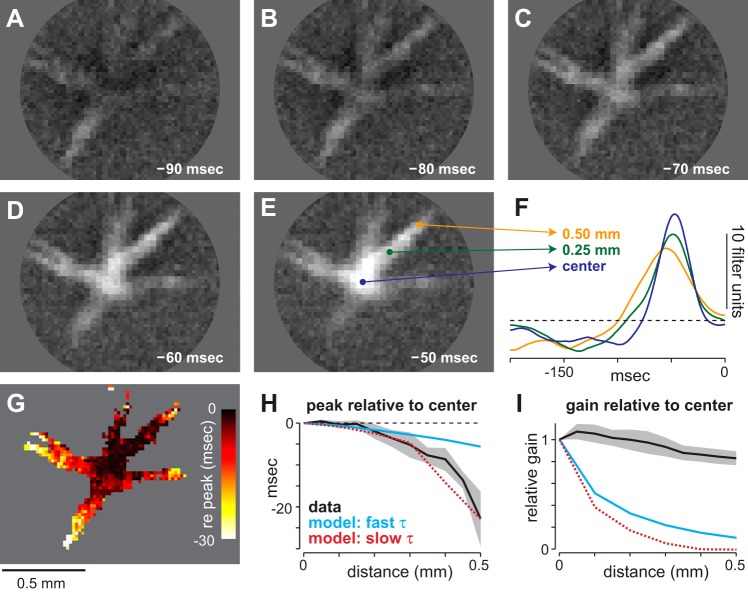
Spatio-temporal dependence of wiry cell receptive fields. *A–E*: slices through the spatio-temporal receptive field at different points in time. *F*: example temporal filters taken from points in the receptive field in *E* as indicated by arrows. *G*: receptive field map of cell in *A–E* in which the peak of each temporal filter is color-coded. *H*: timing of temporal filter peak in dendrites as a function of distance from center. Generally, distal dendrites peaked earlier than proximal dendrites (*n* = 5 cells; mean ± SE). Cyan and red lines show prediction based on passive membrane properties with fast or slow membrane time constants, respectively. *I*: gain of temporal filter in dendrites as function of distance from center for cells in *H*. Cyan and red lines indicate the prediction of the passive models corresponding to *H*.

The spatial receptive fields of wiry cells demonstrated that stimulation over much of the extensive dendritic tree was transmitted to the soma (Fig. 3). In addition to a spatial receptive field map, this analysis provided insight into the spatial dependence of kinetics in wiry cells. This dependence is illustrated by taking sequential slices in time through the spatio-temporal receptive field (Fig. 4, *A–E*). At the earliest time point (−90 ms; Fig. 4*A*), stimulation of the receptive field ∼0.5–0.6 mm from the center produced voltage changes at the soma. This can be visualized in Fig. 4*A* as bright areas in the more distal regions of the receptive field. The receptive field map shifted over time, with bright areas moving progressively toward the center such that the areas most proximal to the recording site were visible only at later time points (Fig. 4, *B* and *C*). Several individual temporal filters were examined for systematic differences between central and more distal regions of the receptive field. Example filters taken from central and more distal regions of the receptive field displayed differences in kinetics, with distal filters peaking earlier than the central filter (Fig. 4, *E* and *F*).

To determine whether this difference in kinetics was systematic and consistent, we calculated the time at which each temporal filter peaked relative to the central filter as a function of distance from the center (Fig. 4*H*). Across cells, the temporal filter peaked earlier at distal positions than at proximal positions (Fig. 4*H*) and the time delays were consistently long for stimuli presented in regions of the receptive field at great distances from the soma. On average, signals originating 0.5 mm away from the center exhibited a lag of ∼22 ms.

In addition, distal filters exhibited a decrease in gain (i.e., signal decay) relative to the center as evidenced by the smaller height of their temporal filters (Fig. 4*I*). Signals originating 0.5 mm away from the center exhibited a decrease in gain/signal of ∼18%.

Are the changes in gain and kinetics for inputs across the dendrites consistent with signal propagation via passive dendritic conductances? To answer this question, we constructed a computational model of a wiry cell based on our somatic and dendritic measurements (NEURON, release 7.3; Hines and Carnevale 1997, 2001). The model contained passive (electrotonic) conductances with parameters taken from previous work on passive dendritic properties (see materials and methods). The model used a membrane time constant (τ) in the lower end of the normal physiological range (τ 16 ms; Spruston et al. 2008; Spruston and Johnston 1992; Stuart and Spruston 1998). However, this passive model was a poor fit for both the time lag and the gain values we measured in wiry cells (Fig. 4, *G* and *H*).

If the parameters of the passive model were inaccurate, this would explain the large difference between the model predictions and data. To test this, we varied the model parameters in order to fit the time course data in Fig. 4*G*. The best fit was achieved by increasing the membrane time constant 25-fold (τ 400 ms), but this value for τ is likely outside of physiological range (Spruston et al. 2008; Spruston and Johnston 1992; Stuart and Spruston 1998). Moreover, while this value produced a better fit for the time course of signal integration (Fig. 4*G*), it predicted gain/signal values that were much lower than our actual measurements (Fig. 4*I*). Thus we interpret this failure of the passive conductance model as further support for active signal propagation in wiry cells.

#### Signals propagated from distal dendrites via active processes.

**Fig. 5.**
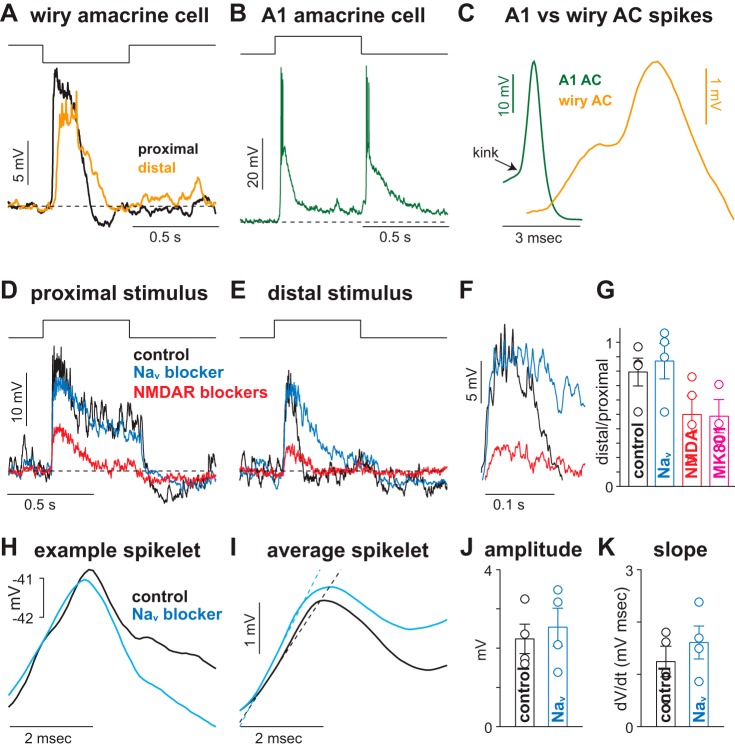
Propagation of signals from distal dendritic processes. *A*: voltage responses of an OFF wiry amacrine cell to a black bar (contrast −100%, width 90 μm, height 270 μm, duration 0.5 s) presented either proximally or distally (0.56 mm) to the soma. *B*: voltage recording from an A1 amacrine cell stimulated with a contrast increment (contrast 100%, duration 0.5 s). *C*: example spikes from an A1 amacrine cell (AC) and a wiry amacrine cell. *D*: responses of an ON wiry cell to a white spot centered on the soma (contrast −100%, diameter 0.52 mm, duration 0.5 s) under control conditions, with voltage-gated Na^+^ (Na_V_) channels blocked (TTX 0.5 μM), and with NMDA spikes blocked (*R*-CPP 15 μM, MK-801 10 μM). *E*: responses of cell in *B* to a white ring centered on the soma (inner diameter 0.78 mm, outer diameter 0.91 mm). *F*: peak responses for conditions in *E*. Spikelets seen under control condition were not suppressed by TTX but were abolished by NMDA receptor antagonists. *G*: response amplitude to the distal ring relative to the proximal spot for the 3 conditions in *D* and *E* averaged across 4 wiry cells and recordings with intracellular MK-801 (1 mM; *n* = 3 cells) (mean ± SE). *H*: example spikelets in the same cell under control conditions and with Na_V_ blockade. *I*: average spikelet across 4 cells for conditions in *H*. Dashed line indicates the fitted slope during the spikelet's initial rising phase. *J* and *K*: spikelet amplitude (*J*) and initial slope of spikelets (*K*) across 4 cells for conditions in *H*. Circles indicate individual cells; error bars indicate SE.

The white noise data predict that signals originating in more distal dendritic processes should propagate well to the soma (Fig. 4*I*). Thus presenting the same stimulus at proximal and more distal portions of the same dendrite should result in comparable responses at the soma. We tested this prediction by stimulating a dendrite adjacent to the soma or at a distance of 0.56 mm away from the soma with a 90-μm-wide bar shown to scale in Fig. 5*A* (duration 0.5 s). The response evoked by distal stimulation was ∼25% smaller than that of proximal stimulation (∼11 mV vs. ∼15 mV). By comparison, the passive conductance model would predict a peak depolarization of <1.5 mV. Thus these results lend further support for active signal propagation in wiry cell dendrites.

These data further underscore the effectiveness of signal propagation in wiry cell dendrites. Our dendritic model suggested that dendritic spikes were the likely biophysical mechanism responsible for effective signal propagation in wiry cell dendrites. Previous work in the retina has found evidence for amacrine cells exhibiting conventional, TTX-sensitive sodium spikes, including A1 amacrine cells in the macaque monkey retina (Bloomfield 1996; Cook et al. 1998; Cook and McReynolds 1998; Dacey 1989; Davenport et al. 2007; Greschner et al. 2014; Protti et al. 2014). However, our recordings from ganglion cells and A1 amacrine cells were qualitatively different from wiry amacrine cells recorded with identical pipettes and intracellular solution. A1 cells exhibited conventional high-amplitude (>25 mV) sodium spikes characterized by a fast rise time and abrupt decay (Fig. 5, *B* and *C*). Conversely, the spikelets recorded from wiry cells were much smaller in amplitude (<3 mV), with slower onset and decay (Fig. 5*C*). Moreover, the pattern observed in wiry cells of high-frequency spikelets riding atop a large depolarizing envelope is characteristic of dendritic NMDA spikes (Augustinaite et al. 2014; Major et al. 2008, 2013; Smith et al. 2013).

If active properties are, indeed, important for signal propagation in wiry cell dendrites, then blocking these active properties should decrease signal transfer from distal dendrites to the soma. One would predict that proximal stimulation would also be affected, but to a lesser extent, as electrotonic spread would be more effective at conveying proximal signals to the soma. We tested for active dendritic processes in wiry cells by comparing proximal and distal stimulation after blocking Na_V_ channels (TTX 0.5 μM) and NMDA spikes (*R*-CPP 15 μM, MK-801 10 μM; Fig. 5, *D–G*) (Augustinaite et al. 2014; Major et al. 2008, 2013; Smith et al. 2013). Under control conditions distal stimulation elicited a depolarization ∼80% of the proximal stimulus (*n* = 4 cells), and blocking Na_V_ channels had little effect on the magnitude of the depolarization (*n* = 4 cells). The distal response was more sustained in the presence of the Na_V_ antagonist—possibly caused by suppressing inhibition from wide-field, spiking amacrine cells (Fig. 5, *E* and *F*). Despite this effect, the putative dendritic spikelets were unaffected during this drug manipulation (Fig. 5*F*) and the relative response of distal-to-proximal stimulation was not significantly different from control (*P* = 0.43). However, blocking NMDA receptors with either bath-applied antagonists (*n* = 4 cells) or intracellular MK-801 (1 mM; *n* = 3 cells) strongly attenuated the responses to both proximal and distal stimulation and suppressed the spikelets. Furthermore, the NMDA receptor antagonists suppressed the distal response more than the proximal response relative to control (Fig. 5*G*; *P* < 0.05).

To determine whether the Na_V_ antagonist attenuated the dendritic spikelets, we calculated the peak amplitude and slope of the initial rising phase for spikelets under control conditions and Na_V_ channel blockade (Fig. 5, *H–K*; *n* = 4 cells). The average spikelet waveform was similar between the two conditions (Fig. 5*I*). Moreover, spikelet amplitude and initial slope were not significantly different between the two conditions (*P* > 0.4 in both cases), which further suggests that Na_V_ channels do not contribute substantially to dendritic spikelets in wiry amacrine cells (Fig. 5, *J* and *K*).

The experiments of Figs. 3–5 show the surprisingly effective propagation of signals from the distal dendrites to the soma of wiry cells. This propagation appears to rely on active dendritic properties, particularly voltage-sensitive NMDA receptors.

#### Wiry cells were agnostic to the direction and sign of moving objects.

If potentials sum along the wiry cell dendrite, the spatio-temporal properties derived from the receptive field mapping predict that wiry cells should depolarize strongly to stimuli moving inwardly and weakly to stimuli moving outwardly.

**Fig. 6.**
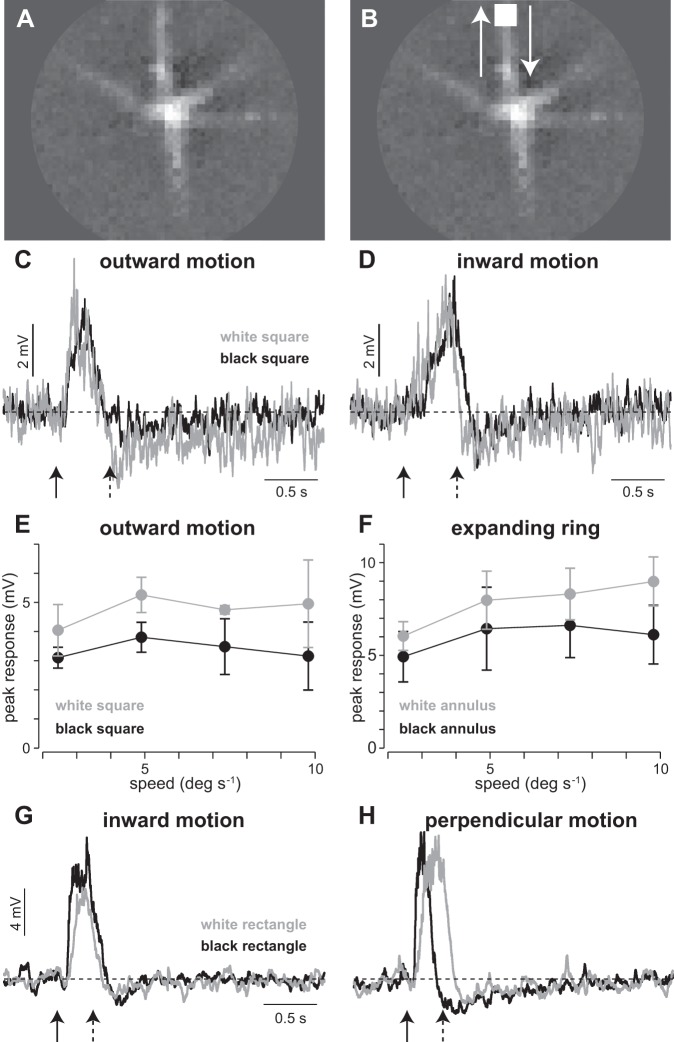
Wiry cells are ON-OFF to moving objects. *A*: spatial receptive field of an ON-type wiry amacrine cell. *B*: schematic showing location of a moving square relative to the stationary receptive field. *C*: responses of cell in *A* to outwardly moving black and white squares. Solid and dashed arrows indicate onset and offset of motion, respectively. *D*: responses of the same cell to inwardly moving black and white squares. *E*: wiry cell responses to outward squares of varying velocity (*n* = 3 cells). *F*: responses to expanding ring at multiple velocities (*n* = 5 cells). *G*: responses of an OFF-type wiry amacrine cell to a rectangle (90 μm × 270 μm; contrast ±100%; speed 1.084 mm/s, 5.42°/s) moving perpendicular to long axis and inwardly along wiry cell's dendrite. *H*: responses of cell in *G* to motion perpendicular to the same dendrite.

To test this prediction, white and black squares were moved (90 μm × 90 μm; contrast ±100%; speed 1.084 mm/s, 5.42°/s) along one of the dendritic processes (Fig. 6, *A* and *B*). The squares either started near the soma and moved outwardly along the dendrite (Fig. 6*C*) or started 0.54 mm away and moved inwardly toward the soma (Fig. 6*D*; solid and dashed arrows indicate onset and offset of motion, respectively). As expected, the cell depolarized after the onset of inward motion of the white square, showing a peak depolarization of ∼5 mV (Fig. 6*D*). However, the outwardly moving square produced a similar depolarization as the inwardly moving square (Fig. 6*C*). Interestingly, the same ON-type wiry cells that hyperpolarized to stationary black spots depolarized to black moving squares (Fig. 6, *C* and *D*). To determine whether this nonlinear response to moving objects was dependent on speed, we repeated the moving square experiment at four speeds between 2 and 10°/s (0.5–2.5 mm/s; Fig. 6*E*). The peak response was similar for the black and white squares across the range of speeds tested. In addition to moving squares, we used expanding or contracting rings to test the response symmetry to white and black moving objects. As with the moving squares, expanding and contracting rings produced similar peak depolarizations for white and black stimuli (Fig. 6*F*).

These results were unexpected, because the linear-nonlinear model and other light responses (Figs. 2–5) predicted that wiry cells should be either ON or OFF, not ON-OFF (Fig. 6). Thus the ON-OFF responses to moving squares and rings may indicate that the functional role of wiry cells is related to encoding of motion. For this to be the case, one would expect that wiry cells should be ON-OFF to motion, not only parallel but also in other orientations relative to their dendrites. Thus we repeated the experiment with motion perpendicular to the dendrite (Fig. 6*H*). As with motion parallel to the dendrite (Fig. 6*G*), perpendicular motion elicited depolarizations to both black and white stimuli. Thus it seems likely that wiry amacrine cells are involved in encoding biological motion in some way.

The similarity in response magnitude for white and black moving objects was observed for both ON (Fig. 6, *C* and *D*)- and OFF (Fig. 6, *G* and *H*)-type wiry cells. This response symmetry might have a simple explanation. The response to a moving square involved two components—a response of one polarity at the square's leading edge and the opposite polarity at the trailing edge. Thus this symmetry might be observed if adaptation were dictating which response dominates. For example, in an ON wiry cell the response to the leading edge might dominate for a white square, but the trailing edge might dominate for a black square if the black region adopted a higher gain. This would also explain the difference in response latency for the two squares (Fig. 6, *C*, *D*, *G*, and *H*).

## DISCUSSION

Our findings provide the first study of the biophysical and light encoding properties of wiry amacrine cells in the macaque monkey retina. Wiry amacrine cell dendrites exhibited active membrane properties, including NMDA spikes, that were critical to signal propagation over large distances (>0.5 mm; Figs. 2–5). Further experimentation revealed that the response polarity of these cells shifted during object motion, from either ON or OFF to ON-OFF (Fig. 6), supporting a possible role in encoding of visual motion.

### 

#### Dendritic signal spread via active membrane properties.

Somatic recording from wiry cells failed to produce direct evidence of conventional, large-amplitude action potentials (Figs. 2 and 5), but stimulation of distal dendrites 0.5–0.6 mm away produced substantial voltage deflections at the soma (Figs. 3 and 4). The most parsimonious explanation for the great breadth of wiry cell receptive fields is that dendritic signals were propagated via active membrane processes. For technical reasons it was not possible to test directly for action potentials along the dendrites. However, results from an anatomically accurate model of wiry cell dendrites with only passive conductances predicted that a dendritic depolarization of 10 mV at a distance of 0.5 mm from the soma would produce a voltage deflection of ∼1 mV at the soma—an ∼90% decrease, yet our measurements found only a ∼18% reduction in gain over the same distance (Fig. 4*I*). The model simulation was run with a dendritic diameter of 0.35 μm, consistent with wiry cell anatomy. Moreover, an anatomically unrealistic dendritic diameter of 65 μm was required to produce a comparable 18% reduction in voltage over a distance of 0.5 mm.

Propagation velocities along the dendrite were also inconsistent with the passive model (Fig. 4). The measured time delays were longer than those predicted for signal propagation via passive, nonregenerative conduction. Nonregenerative potentials integrate locally and spread quickly for short distances along cell membranes because of rapid decay. In contrast, active membrane processes such as spikes propagate in a regenerative manner without loss of amplitude as long as the depolarization spreads to an adjacent region. However, for unmyelinated fibers in particular, the benefits of distance come at the cost of speed. The spreading depolarization involves the opening of voltage-gated channels followed by the flow of ions through those channels. A number of charge carriers have to flow through the channels to change the local membrane potential sufficiently to trigger nearby voltage-gated channels to propagate the signal. Each of the steps takes time, slowing the process relative to electrotonic spread in which the individual charge carriers move relatively small distances with little delay.

#### Signal propagation via NMDA spikes.

The pattern of depolarization in wiry cells was reminiscent of NMDA spikes/plateau potentials that have been observed in other areas of the central nervous system such as the hippocampus (Wei et al. 2001), lateral geniculate nucleus (Augustinaite et al. 2014), primary visual cortex (Smith et al. 2013), and other cortical areas (Major et al. 2008, 2013; Milojkovic et al. 2004, 2005; Schiller et al. 2000). Moreover, our pharmacology experiments indicated that NMDA receptors, but not TTX-sensitive Na^+^ channels, enhanced signal propagation along wiry cell dendritic processes (Fig. 5). Thus NMDA receptors likely produce regenerative potentials—NMDA spikes—in the dendrites. Other voltage-gated channels likely contribute to signal propagation as well, but future studies will be necessary to determine the relative contribution of these mechanisms to dendritic computation in wiry cells.

#### “Wirylike” wide-field amacrine cells in primates and other vertebrates.

Given the stereotyped morphology and stratification pattern, we suggest that the cells described here correspond to the wiry amacrine cells described previously in Golgi studies of macaque (Mariani 1990) and human (Kolb et al. 1992) retina. Interestingly, the aforementioned studies report the somata to be located in the inner nuclear layer, the inner plexiform layer, and the ganglion cell layer. Therefore, it is tempting to speculate that various subtypes of wiry amacrine cells exist, which may stratify in many, if not all, sublaminae of the inner plexiform layer. Such a stratification pattern of this particular group of wide-field amacrine cells has been suggested previously (Masland 2001). Most anatomical data on wide-field amacrine cells were derived from Golgi staining or single-cell dye injections. However, Majumdar and colleagues (Majumdar et al. 2008) were able to analyze a complete population of amacrine cells in macaque retina with a morphological appearance similar to wiry cells. There, the mean distance to the nearest neighbor was extremely small (∼60 μm) considering the enormous size of the cells, with an estimated dendritic spacing of only 4 μm between dendrites of neighboring cells. Therefore, the group of wide-field amacrine cells described here may form dense dendritic networks at various, distinct levels within the retinal neuropil. In addition, morphologically similar amacrine cells are well known from species other than primates [e.g., rabbit (Bloomfield 1994; Bloomfield and Völgyi 2007; MacNeil et al. 1999; MacNeil and Masland 1998), mouse (Knop et al. 2014; Lin and Masland 2006; Pérez De Sevilla Müller et al. 2007; Völgyi et al. 2009), guinea pig (Kao and Sterling 2006), turtle (Ammermüller et al. 1995; Ammermüller and Weiler 1988; Jensen and DeVoe 1982), cat (Wässle et al. 1987), pigeon (Mariani 1982), and carp (Teranishi et al. 1987)]. Thus the widespread existence of “wirylike” amacrine cells suggests that these cells are part of common retinal circuitries, which are conserved between species.

#### Significance for retinal computation.

Here we report the response properties of neurons in the primate retina that are capable of collecting information over vast regions of the visual field, making them appropriate for mediating long-range interactions in vision. Wiry cell receptive fields were measured out to a radius of ∼0.6 mm from the soma, the physical limit of our projection system (Figs. 3–6; see materials and methods). Given the fidelity of signals measured in wiry cell receptive fields at a radius of 0.5–0.6 mm, we would predict that signals would also propagate well from the more distal dendritic processes. This proposition is also consistent with the anatomy of wiry cells—we did not observe obvious anatomical differences between proximal and distal dendrites. This was also true of putative sites of synaptic output—varicosities were located along the entire dendritic length (Fig. 1*E*). On the basis of these observations, we propose that wiry cells integrate over their entire dendritic tree, creating receptive field diameters of ∼2 mm or ∼10° of visual angle. This information from distal regions of visual space would then be conveyed, via output synapses, to postsynaptic neurons along the entire length of the dendritic tree. This suggests that wiry cells would be useful for removing uninformative correlations in the retinal input (Barlow 1961; Field 1987; Laughlin 1981; Pitkow and Meister 2012), size selectivity (Cook and McReynolds 1998; Farrow et al. 2013; Hoggarth et al. 2015), and object motion selectivity (Ölveczky et al. 2003). However, further studies will be necessary to determine the precise role of wiry cells in visual coding.

## GRANTS

This work was supported by the Helen Hay Whitney Foundation, the German Research Foundation (DFG PU 469/2-1), NIH (Grants R01 EY-09303, R01 EY-11850, F32 EY-024507, P30 EY-01730), Research to Prevent Blindness, and the Howard Hughes Medical Institute.

## DISCLOSURES

No conflicts of interest, financial or otherwise, are declared by the author(s).

## AUTHOR CONTRIBUTIONS

Author contributions: M.B.M., C.P., F.R., and J.N. conception and design of research; M.B.M. and C.P. performed experiments; M.B.M. and C.P. analyzed data; M.B.M., C.P., F.R., J.N., and M.N. interpreted results of experiments; M.B.M. and C.P. prepared figures; M.B.M., C.P., F.R., J.N., and M.N. drafted manuscript; M.B.M., C.P., F.R., J.N., and M.N. edited and revised manuscript; M.B.M., C.P., F.R., J.N., and M.N. approved final version of manuscript.

## References

[B1] Ammermüller J, Muller JF, Kolb H The organization of the turtle inner retina. II. Analysis of color-coded and directionally selective cells. J Comp Neurol 358: 35–62, 1995. 7560276 10.1002/cne.903580103

[B2] Ammermüller J, Weiler R Physiological and morphological characterization of OFF-center amacrine cells in the turtle retina. J Comp Neurol 273: 137–148, 1988. 3417900 10.1002/cne.902730202

[B3] Augustinaite S, Kuhn B, Helm PJ, Heggelund P NMDA spike/plateau potentials in dendrites of thalamocortical neurons. J Neurosci 34: 10892–10905, 2014. 25122891 10.1523/JNEUROSCI.1205-13.2014 PMC6705260

[B4] Baccus SA, Ölveczky BP, Manu M, Meister M A retinal circuit that computes object motion. J Neurosci 28: 6807–6817, 2008. 18596156 10.1523/JNEUROSCI.4206-07.2008 PMC6670970

[B5] Barlow HB Action potentials from the frog's retina. J Physiol 119: 58–68, 1953a. 13035717 10.1113/jphysiol.1953.sp004828 PMC1393041

[B6] Barlow HB Summation and inhibition in the frog's retina. J Physiol 119: 69–88, 1953b. 13035718 10.1113/jphysiol.1953.sp004829 PMC1393035

[B7] Barlow HB The coding of sensory messages. In: Current Problems in Animal Behaviour, edited by Thorpe WH, Zangwill OL Cambridge, UK: Cambridge Univ. Press, 1961, p. 331–360.

[B8] Barlow HB, Derrington AM, Harris LR, Lennie P The effects of remote retinal stimulation on the responses of cat retinal ganglion cells. J Physiol 269: 177–194, 1977. 894539 10.1113/jphysiol.1977.sp011898 PMC1283708

[B9] Bloomfield SA Orientation-sensitive amacrine and ganglion cells in the rabbit retina. J Neurophysiol 71: 1672–1691, 1994. 8064341 10.1152/jn.1994.71.5.1672

[B10] Bloomfield SA Effect of spike blockade on the receptive-field size of amacrine and ganglion cells in the rabbit retina. J Neurophysiol 75: 1878–1893, 1996. 8734587 10.1152/jn.1996.75.5.1878

[B11] Bloomfield SA, Völgyi B Response properties of a unique subtype of wide-field amacrine cell in the rabbit retina. Vis Neurosci 24: 459–469, 2007. 17900375 10.1017/S0952523807070071

[B12] Boos R, Schneider H, Wässle H Voltage- and transmitter-gated currents of AII-amacrine cells in a slice preparation of the rat retina. J Neurosci 13: 2874–2888, 1993. 7687279 10.1523/JNEUROSCI.13-07-02874.1993 PMC6576675

[B13] Brenner N, Bialek W, de Ruyter van Steveninck R Adaptive rescaling maximizes information transmission. Neuron 26: 695–702, 2000. 10896164 10.1016/s0896-6273(00)81205-2

[B14] Cembrowski MS, Logan SM, Tian M, Jia L, Li W, Kath WL, Riecke H, Singer JH The mechanisms of repetitive spike generation in an axonless retinal interneuron. Cell Rep 1: 155–166, 2012. 22832164 10.1016/j.celrep.2011.12.006 PMC3406326

[B15] Chichilnisky EJ A simple white noise analysis of neuronal light responses. Network 12: 199–213, 2001. 11405422

[B16] Cook PB, Lukasiewicz PD, McReynolds JS Action potentials are required for the lateral transmission of glycinergic transient inhibition in the amphibian retina. J Neurosci 18: 2301–2308, 1998. 9482814 10.1523/JNEUROSCI.18-06-02301.1998 PMC6792907

[B17] Cook PB, McReynolds JS Lateral inhibition in the inner retina is important for spatial tuning of ganglion cells. Nat Neurosci 1: 714–719, 1998. 10196588 10.1038/3714

[B18] Dacey DM Axon-bearing amacrine cells of the macaque monkey retina. J Comp Neurol 284: 275–293, 1989. 2754037 10.1002/cne.902840210

[B19] Davenport CM, Detwiler PB, Dacey DM Functional polarity of dendrites and axons of primate A1 amacrine cells. Vis Neurosci 24: 449–457, 2007. 17550636 10.1017/S0952523807070010 PMC3130004

[B20] Devries SH, Baylor DA Mosaic arrangement of ganglion cell receptive fields in rabbit retina. J Neurophysiol 78: 2048–2060, 1997. 9325372 10.1152/jn.1997.78.4.2048

[B21] Farrow K, Teixeira M, Szikra T, Viney TJ, Balint K, Yonehara K, Roska B Ambient illumination toggles a neuronal circuit switch in the retina and visual perception at cone threshold. Neuron 78: 325–338, 2013. 23541902 10.1016/j.neuron.2013.02.014

[B22] Field DJ Relations between the statistics of natural images and the response properties of cortical cells. J Opt Soc Am A 4: 2379–2394, 1987. 3430225 10.1364/josaa.4.002379

[B23] Freed MA, Pflug R, Kolb H, Nelson R ON-OFF amacrine cells in cat retina. J Comp Neurol 364: 556–566, 1996. 8820883 10.1002/(SICI)1096-9861(19960115)364:3<556::AID-CNE12>3.0.CO;2-N

[B24] Gentet LJ, Stuart GJ, Clements JD Direct measurement of specific membrane capacitance in neurons. Biophys J 79: 314–320, 2000. 10866957 10.1016/S0006-3495(00)76293-X PMC1300935

[B25] Greschner M, Field GD, Li PH, Schiff ML, Gauthier JL, Ahn D, Sher A, Litke AM, Chichilnisky EJ A polyaxonal amacrine cell population in the primate retina. J Neurosci 34: 3597–3606, 2014. 24599459 10.1523/JNEUROSCI.3359-13.2014 PMC3942577

[B26] Grimes WN, Zhang J, Graydon CW, Kachar B, Diamond JS Retinal parallel processors: more than 100 independent microcircuits operate within a single interneuron. Neuron 65: 873–885, 2010. 20346762 10.1016/j.neuron.2010.02.028 PMC2967021

[B27] Hartline HK The response of single optic nerve fibers of the vertebrate eye illumination of the retina. Am J Physiol 121: 400–415, 1938.

[B28] Hartline HK The receptive fields of optic nerve fibers. Am J Physiol 130: 690–699, 1940.

[B29] Hartveit E Reciprocal synaptic interactions between rod bipolar cells and amacrine cells in the rat retina. J Neurophysiol 81: 2923–2936, 1999. 10368409 10.1152/jn.1999.81.6.2923

[B30] Häusser M, Spruston N, Stuart GJ Diversity and dynamics of dendritic signaling. Science 290: 739–744, 2000. 11052929 10.1126/science.290.5492.739

[B31] Hines ML, Carnevale NT The NEURON simulation environment. Neural Comput 9: 1179–1209, 1997. 9248061 10.1162/neco.1997.9.6.1179

[B32] Hines ML, Carnevale NT NEURON: a tool for neuroscientists. Neuroscientist 7: 123–135, 2001. 11496923 10.1177/107385840100700207

[B33] Hoggarth A, McLaughlin AJ, Ronellenfitch K, Trenholm S, Vasandani R, Sethuramanujam S, Schwab D, Briggman KL, Awatramani GB Specific wiring of distinct amacrine cells in the directionally selective retinal circuit permits independent coding of direction and size. Neuron 86: 276–291, 2015. 25801705 10.1016/j.neuron.2015.02.035

[B34] Ikeda H, Wright MJ Functional organization of the periphery effect in retinal ganglion cells. Vision Res 12: 1857–1879, 1972. 5079253 10.1016/0042-6989(72)90076-4

[B35] Jensen RJ, DeVoe RD Ganglion cells and (dye-coupled) amacrine cells in the turtle retina that have possible synaptic connection. Brain Res 240: 146–150, 1982. 7093712 10.1016/0006-8993(82)90652-7

[B36] Kao YH, Sterling P Displaced GAD65 amacrine cells of the guinea pig retina are morphologically diverse. Vis Neurosci 23: 931–939, 2006. 17266785 10.1017/S0952523806230293

[B37] Knop GC, Pottek M, Monyer H, Weiler R, Dedek K Morphological and physiological properties of enhanced green fluorescent protein (EGFP)-expressing wide-field amacrine cells in the ChAT-EGFP mouse line. Eur J Neurosci 39: 800–810, 2014. 24299612 10.1111/ejn.12443

[B38] Kolb H, Linberg KA, Fisher SK Neurons of the human retina: a Golgi study. J Comp Neurol 318: 147–187, 1992. 1374766 10.1002/cne.903180204

[B39] Kuffler SW Discharge patterns and functional organization of mammalian retina. J Neurophysiol 16: 37–68, 1953. 13035466 10.1152/jn.1953.16.1.37

[B40] Laughlin S A simple coding procedure enhances a neuron's information capacity. Z Naturforsch C 36: 910–912, 1981. 7303823

[B41] Levick WR, Oyster CW, Davis DL Evidence that McIlwain's periphery effect is not a stray light artifact. J Neurophysiol 28: 555–559, 1965. 14328453 10.1152/jn.1965.28.3.555

[B42] Li CY, Zhou YX, Pei X, Qiu FT, Tang CQ, Xu XZ Extensive disinhibitory region beyond the classical receptive field of cat retinal ganglion cells. Vision Res 32: 219–228, 1992. 1574837 10.1016/0042-6989(92)90131-2

[B43] Lin B, Masland RH Populations of wide-field amacrine cells in the mouse retina. J Comp Neurol 499: 797–809, 2006. 17048228 10.1002/cne.21126

[B44] MacNeil MA, Heussy JK, Dacheux RF, Raviola E, Masland RH The shapes and numbers of amacrine cells: matching of photofilled with Golgi-stained cells in the rabbit retina and comparison with other mammalian species. J Comp Neurol 413: 305–326, 1999. 10524341

[B45] MacNeil MA, Masland RH Extreme diversity among amacrine cells: implications for function. Neuron 20: 971–982, 1998. 9620701 10.1016/s0896-6273(00)80478-x

[B46] Major G, Larkum ME, Schiller J Active properties of neocortical pyramidal neuron dendrites. Annu Rev Neurosci 36: 1–24, 2013. 23841837 10.1146/annurev-neuro-062111-150343

[B47] Major G, Polsky A, Denk W, Schiller J, Tank DW Spatiotemporally graded NMDA spike/plateau potentials in basal dendrites of neocortical pyramidal neurons. J Neurophysiol 99: 2584–2601, 2008. 18337370 10.1152/jn.00011.2008

[B48] Majumdar S, Wassle H, Jusuf PR, Haverkamp S Mirror-symmetrical populations of wide-field amacrine cells of the macaque monkey retina. J Comp Neurol 508: 13–27, 2008. 18288700 10.1002/cne.21666

[B49] Mariani AP Association amacrine cells could mediate directional selectivity in pigeon retina. Nature 298: 654–655, 1982. 7099261 10.1038/298654a0

[B50] Mariani AP Amacrine cells of the rhesus monkey retina. J Comp Neurol 301: 382–400, 1990. 2262597 10.1002/cne.903010305

[B51] Masland RH The fundamental plan of the retina. Nat Neurosci 4: 877–886, 2001. 11528418 10.1038/nn0901-877

[B52] McIlwain JT Receptive fields of optic tract axons and lateral geniculate cells: peripheral extent and barbiturate sensitivity. J Neurophysiol 27: 1154–1173, 1964. 14223976 10.1152/jn.1964.27.6.1154

[B53] Meister M, Lagnado L, Baylor DA Concerted signaling by retinal ganglion cells. Science 270: 1207–1210, 1995. 7502047 10.1126/science.270.5239.1207

[B54] Meister M, Pine J, Baylor DA Multi-neuronal signals from the retina: acquisition and analysis. J Neurosci Methods 51: 95–106, 1994. 8189755 10.1016/0165-0270(94)90030-2

[B55] Menger N, Wässle H Morphological and physiological properties of the A17 amacrine cell of the rat retina. Vis Neurosci 17: 769–780, 2000. 11153656 10.1017/s0952523800175108

[B56] Milojkovic BA, Radojicic MS, Antic SD A strict correlation between dendritic and somatic plateau depolarizations in the rat prefrontal cortex pyramidal neurons. J Neurosci 25: 3940–3951, 2005. 15829646 10.1523/JNEUROSCI.5314-04.2005 PMC5643048

[B57] Milojkovic BA, Radojicic MS, Goldman-Rakic PS, Antic SD Burst generation in rat pyramidal neurones by regenerative potentials elicited in a restricted part of the basilar dendritic tree. J Physiol 558: 193–211, 2004. 15155788 10.1113/jphysiol.2004.061416 PMC1664906

[B58] Nelson R, Kolb H A17: a broad-field amacrine cell in the rod system of the cat retina. J Neurophysiol 54: 592–614, 1985. 4045539 10.1152/jn.1985.54.3.592

[B59] Oesch N, Euler T, Taylor WR Direction-selective dendritic action potentials in rabbit retina. Neuron 47: 739–750, 2005. 16129402 10.1016/j.neuron.2005.06.036

[B60] Ölveczky BP, Baccus SA, Meister M Segregation of object and background motion in the retina. Nature 423: 401–408, 2003. 12754524 10.1038/nature01652

[B61] Ölveczky BP, Baccus SA, Meister M Retinal adaptation to object motion. Neuron 56: 689–700, 2007. 18031685 10.1016/j.neuron.2007.09.030 PMC2117331

[B62] Passaglia CL, Enroth-Cugell C, Troy JB Effects of remote stimulation on the mean firing rate of cat retinal ganglion cells. J Neurosci 21: 5794–5803, 2001. 11466451 10.1523/JNEUROSCI.21-15-05794.2001 PMC5130337

[B63] Pérez De Sevilla Müller L, Shelley J, Weiler R Displaced amacrine cells of the mouse retina. J Comp Neurol 505: 177–189, 2007. 17853452 10.1002/cne.21487

[B64] Perry VH, Cowey A The ganglion cell and cone distributions in the monkey's retina: implications for central magnification factors. Vision Res 25: 1795–1810, 1985. 3832605 10.1016/0042-6989(85)90004-5

[B65] Pitkow X, Meister M Decorrelation and efficient coding by retinal ganglion cells. Nat Neurosci 15: 628–635, 2012. 22406548 10.1038/nn.3064 PMC3725273

[B66] Protti DA, Di Marco S, Huang JY, Vonhoff CR, Nguyen V, Solomon SG Inner retinal inhibition shapes the receptive field of retinal ganglion cells in primate. J Physiol 592: 49–65, 2014. 24042496 10.1113/jphysiol.2013.257352 PMC3903351

[B67] Rieke F, Warland D, de Ruyter van Steveninck R, Bialek W Spikes: Exploring the Neural Code. Cambridge, MA: MIT Press, 1997.

[B68] Rodieck RW, Marshak DW Spatial density and distribution of choline acetyltransferase immunoreactive cells in human, macaque, and baboon retinas. J Comp Neurol 321: 46–64, 1992. 1613139 10.1002/cne.903210106

[B69] Schachter MJ, Oesch N, Smith RG, Taylor WR Dendritic spikes amplify the synaptic signal to enhance detection of motion in a simulation of the direction-selective ganglion cell. PLoS Comput Biol 6: 2010. 10.1371/journal.pcbi.1000899 PMC2924322 20808894

[B70] Schiller J, Major G, Koester HJ, Schiller Y NMDA spikes in basal dendrites of cortical pyramidal neurons. Nature 404: 285–289, 2000. 10749211 10.1038/35005094

[B71] Smith SL, Smith IT, Branco T, Häusser M Dendritic spikes enhance stimulus selectivity in cortical neurons in vivo. Nature 503: 115–120, 2013. 24162850 10.1038/nature12600 PMC6319606

[B72] Spruston N, Johnston D Perforated patch-clamp analysis of the passive membrane properties of three classes of hippocampal neurons. J Neurophysiol 67: 508–529, 1992. 1578242 10.1152/jn.1992.67.3.508

[B73] Spruston N, Stuart GJ, Häusser M Dendritic integration. In: Dendrites, edited by Stuart GJ, Spruston N, Ḧausser M New York: Oxford Univ. Press, 2008, p. 351–399.

[B74] Stafford DK, Dacey DM Physiology of the A1 amacrine: a spiking, axon-bearing interneuron of the macaque monkey retina. Vis Neurosci 14: 507–522, 1997. 9194317 10.1017/s0952523800012165

[B75] Stuart G, Spruston N Determinants of voltage attenuation in neocortical pyramidal neuron dendrites. J Neurosci 18: 3501–3510, 1998. 9570781 10.1523/JNEUROSCI.18-10-03501.1998 PMC6793161

[B76] Teranishi T, Negishi K, Kato S Functional and morphological correlates of amacrine cells in carp retina. Neuroscience 20: 935–950, 1987. 3601068 10.1016/0306-4522(87)90254-5

[B77] Völgyi B, Chheda S, Bloomfield SA Tracer coupling patterns of the ganglion cell subtypes in the mouse retina. J Comp Neurol 512: 664–687, 2009. 19051243 10.1002/cne.21912 PMC3373319

[B78] Wässle H, Chun MH, Müller F Amacrine cells in the ganglion cell layer of the cat retina. J Comp Neurol 265: 391–408, 1987. 3693612 10.1002/cne.902650308

[B79] Wei DS, Mei YA, Bagal A, Kao JP, Thompson SM, Tang CM Compartmentalized and binary behavior of terminal dendrites in hippocampal pyramidal neurons. Science 293: 2272–2275, 2001. 11567143 10.1126/science.1061198

[B80] Werblin FS Regenerative amacrine cell depolarization and formation of on-off ganglion cell response. J Physiol 264: 767–785, 1977. 845823 10.1113/jphysiol.1977.sp011693 PMC1307790

[B81] Yamada ES, Dmitrieva N, Keyser KT, Lindstrom JM, Hersh LB, Marshak DW Synaptic connections of starburst amacrine cells and localization of acetylcholine receptors in primate retinas. J Comp Neurol 461: 76–90, 2003. 12722106 10.1002/cne.10672 PMC3342658

[B82] Zaghloul KA, Manookin MB, Borghuis BG, Boahen K, Demb JB Functional circuitry for peripheral suppression in mammalian Y-type retinal ganglion cells. J Neurophysiol 97: 4327–4340, 2007. 17460102 10.1152/jn.01091.2006

